# Plasma cells increased markedly in lymph node in hemophagocytic syndrome: a case report

**DOI:** 10.1186/1757-1626-2-9096

**Published:** 2009-11-27

**Authors:** Li Congyang, Hu Xuexin, Li Hao, Li Chunge, Miao Yingye

**Affiliations:** 1 Department of Pathology, People's Liberation Army 152 hospital, Pingdingshan City, Henan province, PR China; 2 Department of Clinical Laboratories, People's Liberation Army 152 hospital, Pingdingshan City, Henan province, PR China; 3 Department of Pathology, Pingdingshan First People's Hospital, Pingdingshan City, Henan province, PR China

## Abstract

**Introduction:**

Hemophagocytic syndrome is a rare clinicopathological condition characterized by the activation of the mononuclear phagocyte system, resulting in hemophagocytosis in the reticuloendothelial systems. The pathogenesis of HPS remains unclear.

**Case presentation:**

We report the case of a 20-year-old soldier suffering from HPS. Because of long history fever and no reasons being found, his left groin lymph node and left neck lymph node biopsy were done with two weeks interval. We found a marked increase in plasma cells in left neck lymph node during the course of the disease.

**Conclusion:**

Our result provides a new thought for the researchers to understand the mechanisms responsible for the phagocytosis in HPS.

## Introduction

Hemophagocytic syndrome (HPS) is a rare entity characterized by the dysfunction of cytotoxic T cells (CTL) and natural killer (NK) cells, and the activation of the mononuclear phagocyte system [1,2]. Its clinical symptoms include long-term high fever, cytopenias, hepatosplenomegaly, lymphadenopathy, and coagulopathy. HPS is divided into primary and secondary HPS, both of which are trigged by acute infections [3]. The pathophysiology of HPS is very complex, but most researchers now believe that it involves the dysregulation of CTLs and activation of the mononuclear phagocyte system (MPS). In the HPS patient phagocytosis of blood cells is a very complex process and may be related to the production of specific immunoglobulins [1,3-5]. In the current case, we found that plasma cells increased markedly in left neck lymph node during the progression of HPS. This observation supports the idea that antibody responses against blood cells may play an important role in the progression of HPS.

## Case presentation

**Figure 1:**
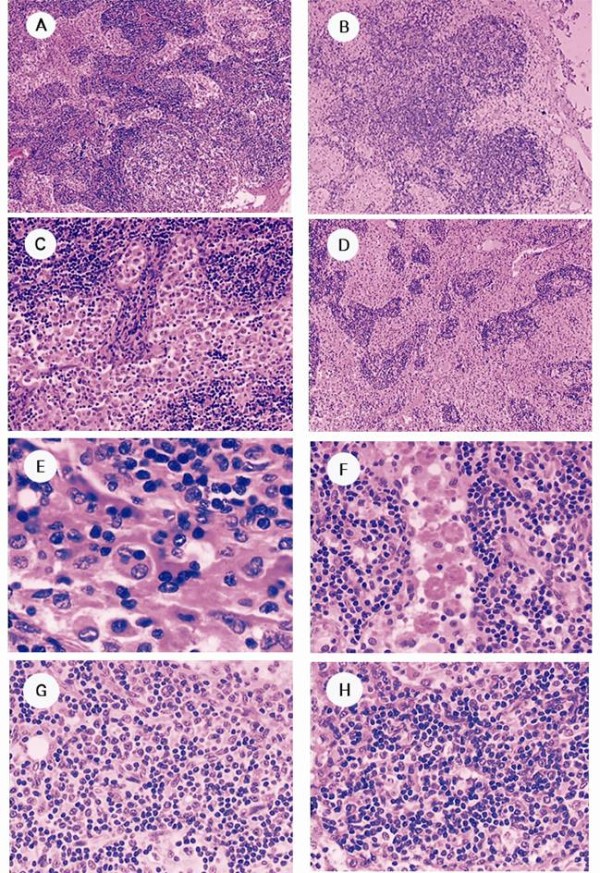
**Hematoxylin and eosin-stained groin and cervical lymph nodes**. A and B, architecture of the groin and cervical lymph nodes, respectively (×40). C and D, sinus expansion and histiocytes in the groin and cervical lymph nodes, respectively (×100). E, a macrophage engulfing a blood cell (×400). F, many macrophages engulfing many blood cells (×400). G, about six plasma cells in the medullary cord of the groin lymph node (×200). H, more than 20 plasma cells in the medullary cord of the cervical lymph node (×200).

A 20-year-old enlisted man presented with a 1-month history of discontinuous fever, panic, and hypodynamia, and was admitted to People's Liberation Army 152 hospital in May 2008. He had no other complaints. The patient had previously been healthy, with no history of infectious diseases or allergies to food or drugs. Physical examination showed a temperature of 37.3°C and no other remarkable signs. Laboratory measurements and examinations revealed no abnormalities. On admission, the patient was administered broad spectrum antibiotics and antiviral drugs. However, his fever failed to resolve and his temperature rose to between 38.5°C and 40.9°C. More than 2 weeks later, lymphadenopathy of the left neck, armpit, and left groin was detected by ultrasound. Left groin lymph node and bone marrow puncture biopsies were performed. Histopathological examination of the groin lymph node showed reactive hyperplasia and revealed one histiocyte engulfing few blood cells (Figure. 1E). The bone marrow smear showed no remarkable results. On admission, we cultured for bacteria, parasites, and fungi, and tested for antibodies to hepatitis A virus (HAV), hepatitis B virus (HBV), hepatitis C virus (HCV), human immunodeficiency virus (HIV), treponema pallidum, chlamydia pneumoniae (CP), mycoplasma (MP), respiratory syncytial virus (RSV), and adenovirus (Adv), however, we were unable to detect the triggering agent. One month later, laboratory tests produced the following values: white blood cell count 1.60 × 10^9^/L, red blood cell 3.42 × 10^12^/L, hemoglobin 107 g/L, platelet count 156 × 10^9^/L, fibrinogen 0.67 g/L, triglyceride 13.9 mmol/L (Table 1). Ultrasound examination revealed hepatosplenomegaly.

**Table 1:** Laboratory findings on admission

Blood cell counts		Coagulation	
WBC	0.80 × 10^9^/L	PT	22.4 sec
RBC	3.42 × 10^12^/L	INR	2.00
HGB	107 g/L	TT	24.2 sec
PLT	156 × 10^9^/L	APTT	42.7 g/L
		FIG	0.67 g/L
			
**Blood chemistry **			
TP	55.50 g/L	CK-MB	32 U/L
ALB	26.60 g/L	HBDH	1929 U/L
GLOB	28.9 g/L	LDH	2467U/L
DB	201.11 μmol/L	TG	13.9 mmol/L
IB	24.73 μmol/L	Urine β_2_-MG	16.5 mg/L
GOT	390 U/L	Blood β_2_-MG	5.8 mg/L
γ-GT	173.00 U/L	CRP	244 mg/L
CK	458 U/L	ALP	208.0 U/L

**Figure 2:**
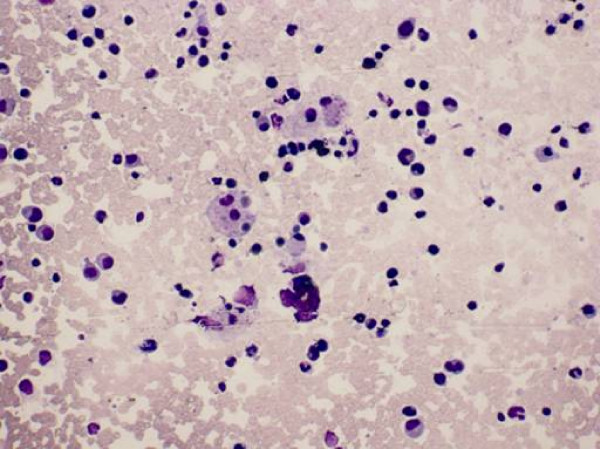
**Bone marrow smear showing macrophages engulfing lymphocytes and red blood cells (Wright's stain ×200)**.

Left neck lymph node biopsy and bone marrow puncture smear were performed. The results were characteristic of HPS (Figure. 1F and Figure. 2). The germinal centers were unclear in both the groin and cervical lymph nodes, and the lymph nodes retained normal structure, except for sinusoidal expansion (Figure. 1A, B). Broadening of the sinuses in the groin lymph node was mainly detected in the medullary area, where they were filled with histiocytes (Figure. 1C). In the cervical lymph node, the subcapsular, peritrabecular and medullary sinuses were expanded and filled with activated histiocytes that were seen to be phagocytosing blood cells (Figure. 1D, Figure 1F). Plasma cell counts averaged 8/high power field (HPF) in the medullary cord of the groin lymph node, while they averaged 17/HPF in the cervical lymph node (Figure 1G, Figure 1H). Treatment was administered according to the Hemophagocytic Lymphohistiocytosis (HLH) 2004 protocol [6], with proper alternation (cyclophosphamide, perarubicin, vincristine sulfate, dexamethasone and etoposide), but the patient suffered multiple organ dysfunction syndrome after therapy, and died from pulmonary infection 2 month later.

## Discussion

HPS is a life-threatening condition characterized by the activation of the MPS. It can be divided into genetic HPS and secondary HPS [2]. Genetic HPS can result from autosomal defects, or can also be associated with immune deficiencies, such as Chediak-Higashi syndrome, X-linked lymphoproliferative syndrome, and Griscelli syndrome. Secondary HPS is associated with exogenous agents (including viruses, bacteria, fungi, parasites, and toxins), endogenous products (including those resulting from tissue damage, metabolic products, free radical stress), rheumatic diseases, and malignant diseases [2,7,8]. In the current case, we were unable to detect the triggering agent, despite culturing for bacteria, parasites, and fungi, and testing for antibodies to HAV, HBV, HCV, HIV, treponema pallidum, CP, MP, RSV, and Adv, and the patient's fever failed to resolve despite treatment with broad-spectrum antibiotics and antiviral drugs. We found no association between drug use and HPS. A diagnosis of lymphoma was eliminated because of the tissue structure and immunoarchitecture.

Although progress has been made in understanding the pathophysiology of HPS, the exact mechanisms responsible for the phagocytosis are unknown in HPS. Researchers found out that there have the RBC antibodies in the EBV-HPS and anti-RBC antibodies were involved in the phagocytosis of RBCs by macrophages [1]. Also several other researchers have demonstrated that virus infection can induce antibody responses against RBCs, platelets, lymphocytes, and endothelial cells [5,9]. As we known the antibodies are produced by the plasma cells. However there has no reports about the exactly changes of plasma cells in HPS patients. In our case the number of plasma cells was markedly increased from 8/HPF in the groin lymph node to 17/HPF in the cervical lymph node during the disease progression. Although we didn't detect changes of special antibodies against blood cells, this observation maybe provide a new idea for researchers to study the pathophysiology of HPS.

## Conclusion

This case report showed plasma cells increased markedly in the left neck node during the progression of HPS. The result maybe provide one way to study the pathophysiology of the HPS.

## Abbreviations

HPS: hemophagocytic syndrome; CTL: cytotoxic T cells; MPS: mononuclear phagocyte system; HAV: hepatitis A virus; HBV: hepatitis B virus; HCV: hepatitis C virus; HIV: human immunodeficiency virus; CP: chlamydia pneumoniae; MP: mycoplasma; RSV: respiratory syncytial virus; Adv: adenovirus; WBC: white blood cell count; RBC: red blood cell; HGB: hemoglobin; PLT: platelet count; PT: prothrombin time; NR: international normalized ratio; TT: thrombin time; APTT: activated partial thromboplastin time; FIG: fibrinogen; TP: total protein; ALB: albumin; GLOB: globulin; DB: direct bilirubin; IB: indirect bilirubin; γ-GT: γ-glutamyl transpeptidase; CK: creatine kinase; CK-MB: creatine kinase-MB; HBDH: hydroxybutyrate dehydrogenase; LDH: lactate dehydrogenase; TG: triglyceride; β2-MG: β2-microglolulin; CRP: C-reactive protein; ALP: alkaline phosphatase.

## Consent

Written informed consent was obtained from the patient' older brother for publication of this case report and accompanying images. A copy of the written consent is available for review by the Editor-in-Chief of this Journal.

## Competing interests

The authors declare that they have no competing interests.

## Authors' contributions

L CY and M YY interpreted the data, operated on the patient and wrote the manuscript. L CG performed the histological examination. H XX reviewed the paper. All authors read and approved the final manuscript.
